# Using Eye Tracking to Examine Effects of Overt Localization on Referential Processing in German Sign Language Sentence Processing

**DOI:** 10.1007/s10936-026-10283-4

**Published:** 2026-06-26

**Authors:** Anne Wienholz, Derya Nuhbalaoglu-Ayan, Nivedita Mani, Annika Herrmann, Markus Steinbach

**Affiliations:** 1 Institute of German Sign Language and Communication of the Deaf, University of Hamburg, Gorch-Fock-Wall 7, 20354 Hamburg, Germany; 2 Department of Romance Languages and Literatures, Goethe-University Frankfurt, Frankfurt, Germany; 3 Georg-Elias-Mueller-Institute of Psychology, Georg-August-University Goettingen, Goettingen, Germany; 4 Department of German Philology, Georg-August-University Goettingen, Goettingen, Germany

**Keywords:** German Sign Language, Eye tracking, Referential processing, Prominence, Referential loci

## Abstract

The processing of referential expressions is influenced by their form, but also by the accessibility of their antecedents. In sign languages, discourse referents are typically assigned to referential loci (R-loci) in the signing space, which can be used to identify discourse referents later on. This study on German Sign Language (DGS) examines whether overt manual localization of a discourse referent with an index sign can be used to increase the prominence and, hence, accessibility of the corresponding discourse referent. Using an eye tracking paradigm, deaf adult DGS signers were presented with DGS videos containing two-sentence discourses introducing two discourse referents in the first sentence. The marking of the two new discourse referents varied by either lack of overt localization or localization of one of the two discourse referents with the index sign. The second sentence started with either the subject or object of the first sentence as a bare noun. Overall, signers fixated the discourse referent that was continued with in the second sentence. However, overt manual localization did not affect signers’ gaze suggesting that overt localization did not lead to facilitatory processing of referential expressions. We speculate that localization may either not carry the linguistic function of enhancing prominence or that the stimuli used in this experiment did not allow for revealing that function.

## Introduction

Sign languages exploit the horizontal plane of the signing space to assign discourse referents to referential loci (so-called R-loci, Liddell, 1990; Lillo-Martin & Klima, 1990; Steinbach & Onea, 2016). In subsequent discourse, these R-loci can be used to establish a relation between a discourse referent and a corresponding anaphoric expression. In many sign languages, the most explicit and unambiguous way to associate a new discourse referent with a specific R-locus is to point with the index finger, i.e., the index sign, towards that location. Alternatively, less explicit means are eye gaze, head movement, body shift or the adaption of the place of articulation for non-body anchored signs, i.e., signs that are produced without contacting the signer’s body (Lillo-Martin, 1986; Winston, 1996). In addition, zero marking is another option in German Sign Language (DGS), which triggers a default assignment of R-loci for the first and second discourse referent to the ipsilateral and contralateral side of the signing space respectively (Steinbach & Onea, 2016; Wienholz et al., 2018; Nuhbalaoglu, 2020). Given the availability and optionality of different more or less explicit strategies of linking new discourse referents to R-loci, the question arises whether the most explicit strategy, i.e., the overt manual assignment of a discourse referent to a specific R-locus with the index sign, increases a discourse referent’s prominence and, thus, affects its accessibility leading to facilitative processing of a subsequent co-referential expression.

In the current study, we investigate the impact of overt localization on the processing of referential expressions in deaf early signers of DGS using an eye tracking paradigm. The outcome of this study does not only contribute to a better understanding of the role of overt localizations of discourse referents during sign language sentence processing but also advances the usability of eye tracking and the visual world paradigm as a method to investigate reference tracking in sign languages. In the following subsection, we first discuss the effect of prominence on referential processing in spoken and sign languages (“Manipulating the Prominence of an Antecedent in Spoken and Sign Languages” section). In the second subsection, we then introduce the main idea and motivation of the current study (“The Current Study” section). The design, procedure and analysis approach are described in more detail in “Methods” section. After presenting the “Results” section, we discuss these results in the context of our hypotheses and the theoretical analysis of the assignment of R-loci in “Discussion” section. The section “Conclusion” closes the paper with some general remarks on R-loci, overt localizations and prominence in sign languages.

### Manipulating the Prominence of an Antecedent in Spoken and Sign Languages

In general, unmarked anaphoric expressions in spoken and sign languages, i.e., anaphoric expressions that are not modified by, for example, adding prosodic cues or a change in word order, are assumed to refer to the discourse referent that is the most activated in the discourse context (Ariel, 1990; Gordon et al., 1993). The comprehension of an anaphoric referential expression is thus not only affected by the form of this anaphoric expression but is also influenced by the accessibility and the prominence of its antecedent. Von Heusinger and Schumacher (2019) characterized prominence of a referent as a dynamic property, which can change due to prominence-lending cues over the course of a discourse. In both modalities, multiple modality-independent factors can serve as such cues, like, for example, order of mention, grammatical function of the antecedent and information structure (for spoken languages, see Ariel, 1990; for sign languages, see Nuhbalaoglu, 2018 and Frederiksen, 2019).

Overall, this suggests that processing effects of prominence are basically universal, but studies hint towards prominence-lending cues as being also modality- and language-specific (Kember et al., 2021). However, compared to spoken languages, there are fewer studies looking at factors affecting prominence and accessibility in sign languages. There is some research that has discussed effects of order of mention (e.g., Wienholz et al., 2023), information structure (for an overview of information structure in sign languages, see Wilbur, 2012 and Kimmelman & Pfau, 2021), prosodic stress (Wilbur, 1999) and the use of focus particles (Herrmann, 2013; Wilbur, 2012). Recently, de Souza Santos (2025) has argued that the spatial person agreement marker (pam, Steinbach, 2022) in DGS can function as an attention-shifting element that shifts the prominence from the subject to the object. Thus, an object marked with pam can be more prominent than the actual subject of the sentence. pam can co-occur with other prominence-lending cues such a change in word order or specific nonmanuals, which might lead to an even higher prominence status of the corresponding discourse referent.

The last example, i.e. the prominence increasing function of the agreement marker pam, shows that not only the information status of the antecedent and the form of the anaphora affects referential processing but also the form of the antecedent itself. One interesting example relevant for the present study is discussed in von Heusinger (2011), who argues that German has two specific kinds of indefinite determiners that can be used to make the corresponding discourse referent more prominent (see also Fodor & Sag, 1982 and Endriss & Gärtner, 2005). Similar to pam in DGS, which has been argued to increase the prominence of the object, the two demonstrative indefinite determiners *dies* (‘this’) and *so’n* (*so ein*, ‘such a’) increase the prominence of the corresponding discourse referent. Unlike the weaker indefinite determiner *ein* (‘a’), which does not increase the prominence of a discourse referent, indefinite demonstratives trigger a facilitative processing of a subsequent co-referential expression (for a similar effect with English *this*, see Ionin, 2006; for experimental evidence, see Deichsel & Heusinger, 2011). Von Heusinger (2011) argues that in the indefinite reading, the demonstratives *dies* and *so’n* can be used to indicate a speaker’s intention to focus the corresponding discourse referent introduced with the indefinite demonstrative. Thus, independent of other factors affecting anaphora resolution, e.g., first mention, subject, topicality or focus, the prominence of a new discourse referent could be manipulated by using different kinds of determiners in languages like in German and English.

**Fig. 1:**
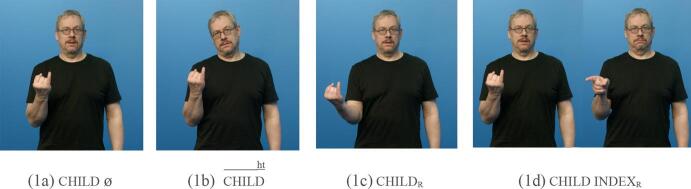
Localization strategies in German Sign Language

As mentioned above, sign languages use the horizontal plane of the signing space in front of the signer to link new discourse referents to R-loci and to use these R-loci in subsequent discourse to re-mention a previous discourse referent. DGS, like many other sign languages, has several options to introduce new discourse referents and link them to R-loci as illustrated in Fig. 1 for the sign child^1^ (Steinbach & Onea, 2016). New discourse referents can be introduced without any overt localization (zero marking) as shown in (1a) for any noun. In this case, the first discourse referent is assigned by a default rule to the ipsilateral side of the signing space and the second one to the contralateral side (Steinbach & Onea, 2016). Alternatively, the noun can be accompanied by additional nonmanual markers (NMMs) such as eye gaze, head tilt or body lean, which specify the R-locus the discourse referent is linked to nonmanually (1b). In addition, the phonological location of a non-body anchored noun can be adapted to the R-locus (1c), i.e., signs usually produced in the neutral signing space are instead produced either at the ipsi- or contralateral side of the signing space, thus linking the noun to a specific R-locus. And finally, the noun can co-occur with a postnominal index sign as illustrated in (1d). The strategies shown in (1) increase in terms of how explicit they are, from the least explicit in (1a) to the most explicit strategy in (1d). Note that the last three (overt) strategies can also be combined leading to more explicit mixed strategies.

The use of an additional sign in an indefinite context, i.e., the manual index sign, is obviously the most explicit and thus the most marked option of linking a new discourse referent to the corresponding R-locus. Since its use is optional with indefinites in DGS, it is a plausible assumption that the index sign might have a similar prominence-enhancing function as demonstrative indefinite determiners in German and English, and can be optionally used to make the new discourse referent more prominent. Consequently, like indefinite demonstratives, the index sign could be used to express the intention of the signer to raise the attention to a unique referent by pointing to the specific R-locus this discourse referent is linked to. The overt pointing to a R-locus in an indefinite context would thus trigger a higher discourse prominence of the corresponding discourse referent.

Interestingly, it has been argued already for some sign languages that the overt localization of a new discourse referent with the index sign can be used to increase the prominence of this discourse referent (Engberg-Pedersen, 1993 for Danish Sign Language; Rinfret, 2009 for Quebec Sign Language; Winston, 1995 for American Sign Language; for an overview, see Barberà, 2021). This observation is supported by the results of a production study conducted by Frederiksen and Mayberry (2022), who asked signers of American Sign Language (ASL) to repeat sentence prompts that did and did not contain overt localization of discourse referents. The results show that, for prompts without overt localization, i.e., fingerspelled names, signers frequently added localization (manual and/or nonmanual) to discourse referents that were re-mentioned subsequently, often using pronominal forms. Unfortunately, the authors provide neither clear frequency data nor a discourse semantic analysis of this specific function of additional manual and nonmanual localizations. Therefore, it remains unclear how often a manual index sign was used for localization and for which specific function the index sign was used. Nevertheless, these observations support the idea that overt localization could carry a prominence-lending function.

Further evidence for the analysis of the indefinite use of the index sign in some sign languages as an indefinite demonstrative is provided by the origin of the index sign itself. Just like the indefinite demonstratives in English or German discussed above, the index sign has developed from a demonstrative and similar to indefinite demonstratives in spoken languages, the demonstrative property of the index sign, i.e. shifting the attention to a unique referent, is retained in the indefinite use of the index sign (for the grammaticalization of pointing signs from pointing gestures, see McBurney, 2004; Pfau & Steinbach, 2011; Pfau, 2011; for differences between pointing signs and pointing gestures, see Fenlon et al., 2019).

Taken together, these observations suggest that the use of the index sign with new discourse referents functions to increase the prominence of the corresponding discourse referent. To the best of our knowledge, no experimental study so far has investigated whether the index sign in sign languages does indeed increase the prominence of the item they accompany and, thus, enhance its accessibility during processing. In this study, we aim to investigate whether the index sign can be used to shift the attention to a unique referent. The study reported in this paper is, thus, the first experimental study that investigates the discourse semantic impact of the index sign on sign language processing.

### The Current Study

The current study examines the influence of overt localization on the processing of discourse referents in DGS. Specifically, we asked whether overt localization of a discourse referent facilitates the processing of that same referent when it is re-mentioned subsequently. To answer this question, we used a visual world eye tracking paradigm, a well-established approach in spoken language research (Huettig et al., 2011). Previous work on sign languages has applied this paradigm as well to examine effects during sign language processing using various stimulus materials (isolated signs: e.g., Lieberman et al. (2015); carrier phrases: e.g., Thompson et al. (2013); question-answer structures: e.g., Wienholz and Lieberman (2019) and sentences: e.g., Wienholz et al. (2021); Wienholz and Lieberman (2025). We will extend this work by using linguistic material that includes two related sentences.

Similar to indefinite demonstratives like *this* in English and *dies/so’n* in German, we expected overt manual localization with the index sign in DGS to increase the prominence of the corresponding discourse referent to, consequently, make it more accessible. We recorded participants’ eye movements while they were simultaneously presented with a DGS video containing a two-sentence discourse and pictures representing the referents mentioned in the discourse. The two-sentence discourses introduced two discourse referents in the first sentence and varied whether and which one of these referents, i.e., either subject or object, were overtly localized, i.e., assigned to a R-locus with the index sign (see Appendix A for video stills, online on OSF (https://osf.io/5vubm/?view_only=e419085fd83748c5a5296318550c7109 [10.17605/OSF.IO/5VUBM]). The second sentence always started with one of the two discourse referents introduced in the first sentence by repeating the respective noun. Thus, the short discourses continued either with the previous subject or object referent. If overt localization using an index sign functions as a prominence cue, we would expect a facilitation effect reflected in more looks to the respective referent when localization and continuation type are matching and are referring to the same referent, i.e., more looks to the subject referent when the subject was localized and continued with at the beginning of the second sentence and more looks to the object referent when the object was localized and re-mentioned in the second sentence. Additionally, we would predict an interference effect when localization and continuation are not matching, i.e., when the subject was localized in the first sentence, but the second sentence continues with the object referent or when the object was localized but the second sentence begins with the subject referent. In contrast, if the overt localization of a discourse referent does not affect processing, we would expect no difference in looking behavior when comparing localized and non-localized referents.

## Methods

### Participants

In total, 24 deaf early signers of DGS (12 female, 12 male) volunteered to participate and were compensated for their time. The participants had a mean age of 34 years (age range: 20–58 years) and had at least high school education level. All participants had deaf parents. Twenty-two participants were exposed to DGS before the age of 5 years. The remaining two participants were exposed to DGS at the age of 8 and 15 years but had access to another sign language before and had been using DGS as their primary language for more than 20 years. Excluding these participants from the analyses did not lead to changes in the results and, therefore, they were included in the analyses. Two additional participants were tested but had to be excluded due to failure of calibrating the eye tracker. All participants were informed about the procedure and gave written consent prior to participation. The Ethics Committee of the Institute of Psychology at the University of Goettingen provided ethics approval for this study.

### Material

The stimulus material consisted of videos containing DGS sentence pairs and pictures. The videos had a size of 732 × 550 pixels and a light grey background. The pictures were color photo-realistic pictures with a size of 300 × 400 pixels and a light grey background. The pictures were presented above the video to ensure that pointing signs in the video were not interpreted as directional pointing towards one of the pictures (Wienholz & Herrmann, 2023). The DGS videos contained sentence pairs consisting of two sentences of the following structure (a full list of the stimulus material is given in Appendix B, online on OSF (https://osf.io/5vubm/?view_only=e419085fd83748c5a5296318550c7109 [10.17605/OSF.IO/5VUBM]). The first sentence, containing three to five signs, introduced two human discourse referents combined with a transitive non-localizing verb in neutral SOV word order (Proske, 2022). Each sentence occurred in four conditions (Table 1), which varied in the presence of overt localization either of the two discourse referents. Localization was established by the index sign which assigned a discourse referent either to an R-locus on the horizontal plane of the signing space. In the *no localization* condition, i.e., the baseline condition, overt localizations of both referents were absent. In the *subject localization* condition, only the subject was localized while the object was not overtly localized. In the *object localization* condition, only the object was localized. For a fully balanced design, subject and object were both localized in the *localization both* condition. However, since we are interested in the effect when adding localization to one of the two referents, this condition was excluded from the analysis and, therefore, will not be further discussed here. Following Steinbach and Onea (2016) and Wienholz et al. (2018), subjects were assigned to the ipsilateral/right side of the signer while objects were localized on the contralateral/left side of the signer. For the discourse referents, we used bare nouns denoting either generic categories (e.g., woman), job descriptions (e.g. cook), relatives (e.g., grandma), or story characters (e.g., witch). No compound signs or proper names were used due to their more complex structure, which might lead to an increased processing load and, thus, might affect the looking behavior of participants. Each noun occurred as subject and object in the first sentence with a maximum occurrence of eight times across all stimuli, i.e., four times as subject and four times as object. Additionally, each noun combination, i.e., combination of subject and object, only occurred once. As verbs, we used transitive spatial and reciprocal verbs signed in the neutral space in front of the signer to avoid any agreement relation between the verb and its localized discourse referents. The three lexical signs, i.e., the two referents and the verb, were kept stable across all conditions for each sentence pair.

**Table 1:** Example stimuli for each combination of continuation type, localization condition and match/mismatch condition

Continuation type	Condition	Match/mismatch	Example stimulus
Subject	No localization (baseline)	None	teacher __ girl __ talk. teacher different city born. ‘A teacher talks with a girl. The teacher was born in a different city.
	Subject localization	Match	teacher ix_r_ girl __ talk. teacher different city born. ‘A teacher talks with a girl. The teacher was born in a different city.
	Object localization	Mismatch	teacher __ girl ix_l_ talk. teacher different city born. ‘A teacher talks with a girl. The teacher was born in a different city.
Object	No localization (baseline)	None	cook __ woman __ meet. woman a-lot eat can. ‘A cook meets a woman. The woman can eat a lot.’
	Subject localization	Mismatch	cook ix_r_ woman __ meet. woman a-lot eat can. ‘A cook meets a woman. The woman can eat a lot.’
	Object localization	Match	cook __ woman ix_l_ meet. woman a-lot eat can. ‘A cook meets a woman. The woman can eat a lot.’

The abbreviation ix represents the index sign that was either produced on the right side (ix_r_) or on the left side (ix_l_) of the signer

The second sentence of each stimulus contained four signs and always started with a full noun corresponding to one of the two referents introduced in the first sentence, resulting in two continuation types, followed by three additional signs (Table 1). In the subject continuation type, the first sign was the subject of the first sentence (e.g., teacher) while in the object continuation type, the object of the previous sentence (e.g., woman) was presented as the first sign of the second sentence. We opted for a full noun instead of a pronoun because the localizing index sign and the pronoun use the same form, i.e., pointing with an extended index finger. Therefore, it is hard to ensure that different functions of the same sign are activated in those contexts (please be referred to the discussion section where we discuss potential problems of this methodological choice). In contrast, the full noun is unambiguous. The signs following the first sign were used maximally twice and were as neutral as possible with respect to the two discourse referents. The second sentence was always syntactically, and, more importantly, also semantically well-formed irrespective of which discourse referent was re-mentioned in the second sentence.

Additionally, combining the factors localization and continuation type has led to *match* and *mismatch* conditions (Table 1). In the match conditions, localization with the index sign in the first sentence and continuation type in the second sentence apply to the same referent. Thus, match conditions either localize the subject in the first sentence and continue with the subject in the second sentence or localize the object in the first sentence and then continue with the object in the second sentence. In contrast, the mismatch conditions contain a mismatch between the referents to which localization and continuation type apply. Accordingly, mismatch occurs when the subject is localized in the first sentence but the second sentence continues with the object or when the object is localized and is continued with the subject in the second sentence.

In total, 320 sentence pairs were constructed with 40 sets in each condition for each of the two continuation types. One sentence pair was excluded before running the experiment due to an error in one of the stimulus videos so that 316 sentence pairs remained for testing. An additional set of six practice sentence pairs with the same structure were constructed with three sets for each continuation type and one pair for each condition. Nouns and verbs used in the practice items were not used in the stimulus material.

The sentence pairs were constructed, discussed and recorded with the help of a deaf signer of DGS, i.e., the signer was born deaf and grew up in a deaf signing family. Videos were recorded using a Sony HDR-CX550VE camcorder. The signer was instructed to slow down the speed of signing and to keep a longer final hold of the referent sign in case of an absent index sign to keep the overall length of the corresponding constituent and sentence pair more or less similar across conditions. The first sentence of each stimulus ended with a final hold of the verb, followed by a natural prosodic break before the start of the second sentence. Since we do not know enough about the influence of nonmanuals on sign language processing in general and the processing of discourse referents, the signer only used natural mouthing and lexically specified nonmanuals to produce sentences as natural as possible and ensure lexical understanding, but avoided other nonmanuals, such as eye gaze localization of the referents, brow raise or any additional head and body movement. After recording, videos were cut and processed using the video editing software Adobe Premiere Pro CS6 so that the signer remained motionless for 1000 ms at the beginning and for 1500 ms at the end of each video. Moreover, the background color of each video was changed to a grey tone that was the same as in the presented picture stimuli to avoid any influence from different background colors. Videos were not modified further. Videos had a mean duration of 12,667 ms (range: 8960–18000 ms). An ANOVA revealed a main effect for continuation type (*F*(1, 231) = 44.89, *p* <.001, *β*^*2*^ = 0.16) and condition (*F*(2, 231) = 7.87, *p* <.001, *β*^*2*^ = 0.06), but no interaction (*F*(2, 231) = 0.71, *p* =.492, *β*^*2*^ = 0.01). Follow-up tests showed that videos in the subject continuation type (*M* = 12167 ms) were shorter than in the object continuation type (*M* = 13155 ms). The effect of condition was driven by the difference between the no localization condition (*M* = 12334 ms) and the object localization condition (*M* = 13046 ms). The subject localization condition (*M* = 12621 ms) did not differ significantly from the other conditions.

The picture stimuli represented both referents introduced in a sentence pair shown in the video, e.g., for the object continuation set in Table 1, the picture of a cook and a woman were presented along with each sentence. Pictures were chosen based on the results of a picture-naming task conducted with German native speakers in an online questionnaire. Using Google Forms (Google Inc.), 216 pictures were presented, divided into four lists of 54 pictures each. Note that due to the parallel preparation of a different eye tracking study, pictures from both studies were combined and presented in the same picture-naming task so that pictures of objects and humans were mixed, avoiding biases towards strategic naming. Two pictures were presented for each noun, but in different lists, allowing us to choose the most fitting picture to be used in the final stimuli. Overall, 101 participants (79 female, 22 male, mean age: 25 years) named the presented pictures with one word. To exclude potential effects of culturally shaped concepts and associated labels thereof, eight participants were excluded prior to analysis since they indicated another language than German as their first language in the demographics. During evaluation, the answers to both pictures for each noun were compared and the picture with the most correct namings was chosen for the final stimulus set. In case of equal number of correct namings, the picture was chosen randomly. The selected pictures had a mean naming accuracy of 86.5% (range: 66% − 100%).

### Procedure

Prior to testing, participants were informed about the procedure and the eye tracking paradigm, filled in a background questionnaire and gave written consent. To ensure full understanding, all information was presented in DGS and in written German. Participants were informed about the aim of the study only after the experiment so that they were naïve to the topic during the study.

For stimulus presentation, the sentence pairs were divided into four different lists so that each list only contained one sentence from each set. In total, each list contained 80 stimuli consisting of 40 pairs in the object continuation and 40 in the subject continuation type. For each continuation type, 10 sentence pairs of each of the four conditions were presented with counterbalanced picture positions, i.e., the alignment of the pictures was counterbalanced with respect to the side that a referent was localized at in the video. Alignment refers to the position of the pictures in relation to the order of occurrence of the corresponding referents in the video. For instance, in a sentence from the subject localization condition, the subject was overtly localized on the right side of the signer, which corresponds to the left side of the screen from the perceiver’s perspective. Thus, if the picture of the subject referent was aligned with the localization of that referent in the video, it occurred on the left side of the screen as well. In contrast, the picture of the subject referent was presented on the right side of the screen when it was not aligned to the video. Due to counterbalancing of the picture position, each list had to be presented with the reversed picture position as well, which resulted in eight different lists. Order of trials was randomized on-line by the experimental software with maximally two consecutive trials from the same condition. As mentioned above, due to an error in the video, one sentence pair was excluded with all its four conditions prior to testing and after compiling the randomization lists. Therefore, the final lists each contained 79 stimuli.

Participants were seated at a table in front of a 17-inch computer screen with a resolution of 1280 × 1024 pixels. Their task was to look at the pictures and video presented on the screen. As an explicit task is not required in eye tracking studies, participants did not have to complete an additional explicit task. There are numerous previous eye tracking studies on spoken language that did not apply an explicit task (e.g., Altmann & Kamide, 2009 or Knoeferle et al., 2011 among others) Nevertheless, additional tasks can be combined with eye tracking to obtain reaction times or accuracy data alongside the observation of unrestricted eye movements. To minimize head movements, they were instructed to place their head on a chinrest that was adjusted to a comfortable height for each participant individually. The eye tracker was adjusted manually to capture the participants’ eye with a distance of 50–70 cm from the face. All stimuli were presented using a PC computer and the Eyelink Experiment Builder Software, Version 1.10.165 (SR Research Ltd.). The experiment started with an introduction video in DGS explaining the procedure followed by the practice items to familiarize participants with the eye tracking paradigm and the structure of the experiment. After the practice session was completed and participants’ remaining questions were clarified, the experiment started. In total, 79 trials were presented divided into three blocks (block 1: 30 trials; block 2: 29 trials; block 3: 20 trials) with breaks in-between so that participants could relax their eyes and remove their head from the chinrest. Before each block, a nine-point calibration sequence was presented and before each trial, an additional one-point drift correction, manually accepted by the experimenter, was performed.

**Fig. 2:**
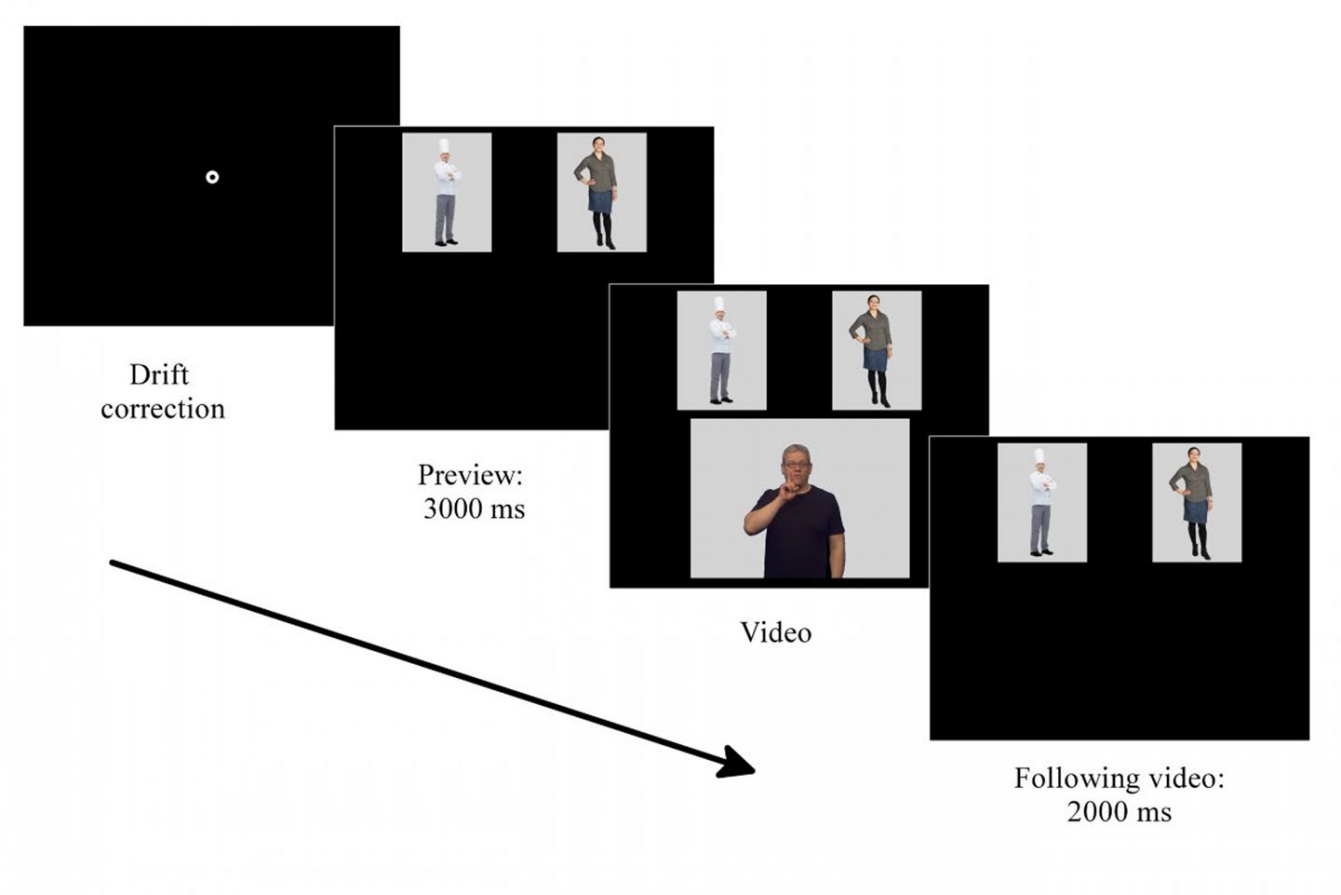
Structure of a single trial

Following the accepted drift correction, a trial continued with the presentation of the picture stimuli presented at the top of the screen for 3000 ms (Fig. 2). In contrast to spoken languages where the linguistic input and the pictures are perceived through different channels (visual and auditory), signers perceive this input through the same visual channel. Therefore, the preview period allows participants to familiarize themselves with the presentation of the information on the screen. After that, the video appeared at the bottom of the screen, while the two pictures stayed in place, and disappeared after the signer finished signing, i.e., when the hands reached their resting position at the side of the signer. The pictures remained on the screen for an additional 2000 ms. Then, the next trial started automatically. Overall, the study took about 20–25 min to complete.

Eye movements were tracked and recorded with a sampling rate of 500 Hz using an EyeLink 1000 Desktop Mount (SR Research Ltd.) with a remote eye tracker. Data for each trial were recorded starting with the presentation of the pictures until they disappeared after the video presentation. Afterwards, the data were binned off-line into 30 ms sequences.

### Analysis Approach

All analyses were done using R (Version 4.3.0, R Core Team, 2023) with the packages eyetrackingR (Forbes et al., 2021), lme4 (Bates et al., 2015) and sjPlot (Lüdecke, 2023) to generate model outputs as well as ggplot2 (Wickham, 2016) and ggsignif (Ahlmann-Eltze & Patil, 2021) to create data visualizations. The condition *both localization* was excluded from the analyses. During data cleaning, we excluded trials that exceeded the trackloss threshold of 25%, resulting in the removal of 34 trials (2.5%).

Signers perceive the linguistic input and the picture through the same visual channel. Initially, they predominantly fixate the video because it contains the linguistic information. Nevertheless, as demonstrated in previous eye tracking studies (e.g., Lieberman et al., 2018; Wienholz & Lieberman, 2019), signers divide their attention between the video and the pictures presented on the screen, i.e., signers might shift back-and-forth between video and pictures. However, when and how often signers shift away from the video is licensed by the structure of the linguistic material. Structures containing semantically “empty” function words provided opportunities for the signers to shift away from the video. If structures do not contain this kind of signs, signers will keep fixating the video to not miss out on the content provided in the video. This behavior has implications for data analysis as traditional analyses approaches from eye tracking studies on spoken languages cannot be easily applied to sign languages.

In the current study, eye movement data were analyzed according to fixations to three areas of interest, i.e., the video and the two pictures, excluding non-AOI looks. We included a time course and a time window analysis. As we are interested in the effect of localization on subsequent referents, and to account for the differences of video length, the time course analysis only included fixations starting at the onset of the second sentence, i.e., when one of the previous referents is re-mentioned, for 1000 ms. This length was selected based on the mean length of the referents (*M* = 514 ms) rounded up to reach 1000 ms. We ran permutation-based analyses (Maris & Oostenveld, 2007) for different comparisons explained in more detail in the results section below. The window analyses included overall fixations in an 800 ms time window by excluding the first 200 ms after the onset of the second sentence to ensure that gaze shifts were in response to the referential expression. We calculated gaze fixations by using log transformations (log[(proportion subject looks + 1]/(proportion object looks + 1)]), which reduced noise, i.e., looking offscreen or to the video, and allowed us to assess participant’s looks to the subject relative to the object. Transformed positive values indicated more looks to the subject while transformed negative values indicated more looks to the object. We ran different mixed-effects models to test for effects of localization, continuation type and match/mismatch with all models containing participants and items as random effects. For these models, we performed a sensitivity analysis using G*Power (Faul et al., 2009). This analysis allowed us to determine the reliable effect size that our study could detect for a given alpha error probability of 0.05 and a power of 80% with our given sample size.

## Results

**Fig. 3:**
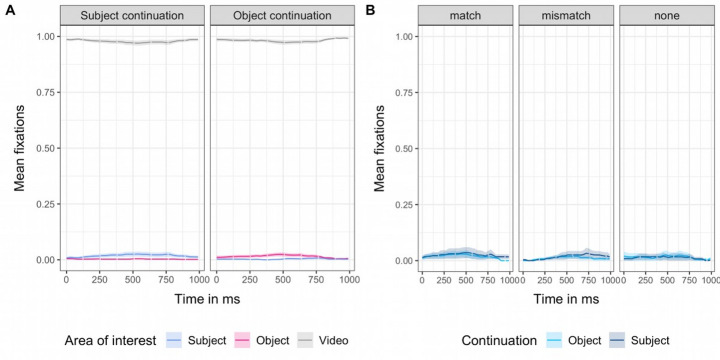
**a** Time course of mean fixations in the subject (left column) and object continuation type (right column) to the video (grey), the subject picture (blue) and object picture (pink) from onset of the second sentence and **b** time course of mean target fixations averaged for the match (left column) and mismatch (middle column) conditions and the no localization condition (right column), in the subject (dark blue) and object continuation type (light blue)

First, we assessed effects of localization on gaze behavior across the time course. We plotted fixations to the video and the two pictures for each continuation type across conditions (Fig. 3a). Visual inspection suggested a low proportion of fixations to the pictures, which is expected since signers are initially fixating the video to perceive the linguistic input, as shown in previous eye tracking studies (e.g., Lieberman et al., 2018, 2022; Wienholz & Lieberman, 2019; among others). Cluster permutation comparing looks to subject and object in each continuation type showed no effect in the subject continuation type and only a marginal significant effect in the object continuation type (*p* =.082) between 270 ms and 540 ms. The same analyses done separately for each localization condition in each continuation type did not reveal any significant divergences (see Appendix C for more details, online on OSF (https://osf.io/5vubm/?view_only=e419085fd83748c5a5296318550c7109] [10.17605/OSF.IO/5VUBM]). Next, we aimed to determine whether gaze patterns are affected when localization is added after a referent. We compared target looks in each of the localization conditions with the baseline where no localization was included, e.g., comparing the subject localization condition to the no localization condition. Target looks are defined based on the continuation type so that in the subject continuation type, the target is the subject and, accordingly, the object is the target in the object continuation type. This analysis showed no significant divergences (see Appendix D for more details, online on OSF (https://osf.io/5vubm/?view_only=e419085fd83748c5a5296318550c7109] [10.17605/OSF.IO/5VUBM)]. Finally, we compared target looks for continuation type in the match and mismatch conditions as well as in the baseline (Fig. 3b). Again, cluster permutation revealed no significant divergences.

**Fig. 4:**
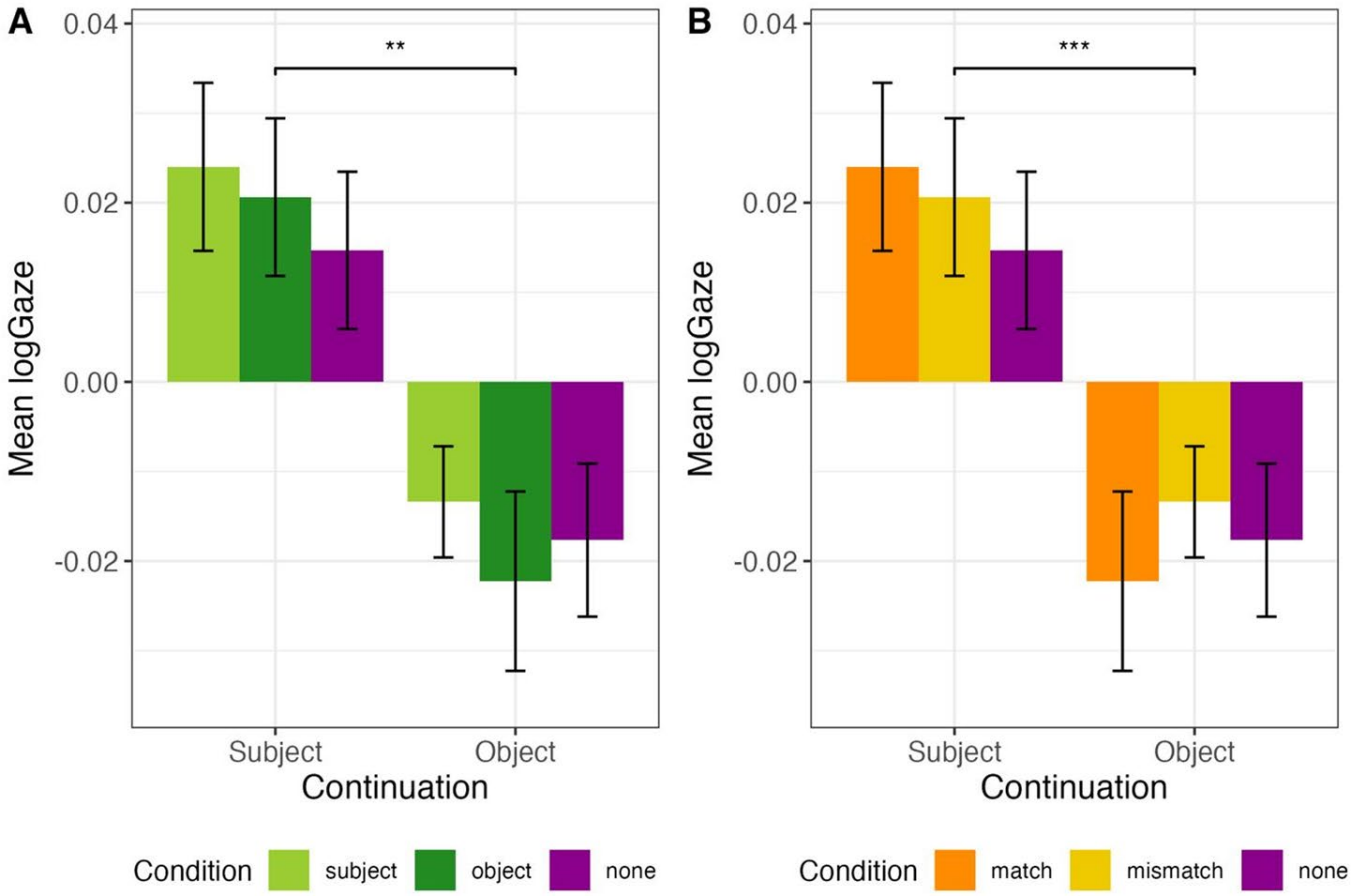
Mean logGaze of subject versus object fixations for the subject (three left most bars) and object continuation type (three right most bars) for **a** the localization conditions none (purple), subject localization (light green) and object localization (dark green) and **b** the recoded conditions match (orange), mismatch (yellow) and none (purple)

Next, in the window analysis, we compared subject to object looks by calculating mean log gaze ratios for the subject picture relative to the object picture (Fig. 4a). We used a mixed-effects model to test for effects of localization and continuation type as well as their interaction, and included participants and items as random effects. Analyses revealed an effect of continuation type, but no effect of localization and no interaction of both factors (Table 2). Subsequently, we ran separate mixed-effects models for the subject continuation type and the object continuation type including the fixed effect of localization and random effects for participants and items. None of the models showed an effect of localization (all *p* >.3). The sensitivity analysis revealed a detectable effect size of *f*^*2*^ = 0.46.

**Table 2:** Window analysis model output: Parameter estimates of the mixed-effects model of effects of continuation type and condition on the proportion of subject vs. object fixations (logGaze transformed). Significant effects are marked in bold.

Predictors	logGaze		
	Estimates	CI	*p*
(Intercept)	−0.02	−0.03 – −0.00	**0.043**
continuation type	0.03	0.01–0.06	**0.008**
object localization condition	−0.00	−0.03–0.02	0.711
subject localization condition	0.00	−0.02–0.03	0.729
continuation type × object localization	0.01	−0.02–0.04	0.545
continuation type × subject localization	0.01	−0.03–0.04	0.771
**Random Effects**			
σ^2^	0.02		
τ_00 item_	0.00		
τ_00 id_	0.00		
N _id_	24		
N _item_	237		
Observations	1388		
Marginal R^2^/Conditional R^2^	0.020/NA		

With respect to the match and mismatch conditions (Fig. 4b), we ran a mixed-effects model testing for effects of match/mismatch condition and continuation type as well as their interaction with participants and items as random effects. The factor match/mismatch was contrast-coded using the no localization condition as the baseline. Analysis showed an effect of continuation type, but no effect of match/mismatch and no interaction (Table 3). The sensitivity analysis revealed a detectable effect size of *f*^*2*^ = 0.46.

**Table 3:** Window analysis model output: Parameter estimates of the mixed-effects model of effects of continuation type and match/mismatch condition on the proportion of subject vs. object fixations (logGaze transformed). Significant effects are marked in bold.

Predictors	logGaze		
	Estimates	CI	*p*
(Intercept)	−0.02	−0.03 – −0.01	**< 0.001**
continuation type	0.04	0.02–0.05	**< 0.001**
match	−0.00	−0.03–0.02	0.711
mismatch	0.00	−0.02–0.03	0.729
continuation type × match	0.01	−0.02–0.05	0.424
continuation type × mismatch	0.00	−0.03–0.04	0.923
**Random Effects**			
σ^2^	0.02		
τ_00 item_	0.00		
τ_00 id_	0.00		
N _id_	24		
N _item_	237		
Observations	1388		
Marginal R^2^/Conditional R^2^	0.020/NA		

## Discussion

The current study investigated the impact of overt manual localization of new discourse referents on the processing of subsequent co-referential anaphoric expressions. We recorded participants’ eye-movements while presenting a video with two-sentence discourses containing two referents and two pictures representing both referents on a screen. Within these discourses, the first sentence introduced two discourse referents either with or without overt localization, i.e., one of the referents or none was overtly localized with an index sign. The subsequent sentence re-mentioned one of the two discourse referents introduced in the first sentence with a bare noun. Overall, the results showed that signers fixated the matching picture of the referent that was re-mentioned. Thus, signers fixated the subject more when the second sentence continued with the subject referent and, accordingly, fixated the object more when the object referent was continued with in the second sentence. However, signers’ gaze patterns were not affected by overt manual localization of the referents, suggesting that the overt introduction of an R-locus with an index sign does not lead to facilitatory processing of the discourse referent that has been linked to an R-locus.

This was the first eye tracking study on sign languages involving signed stimulus material with more than one sentence. The observed effect of continuation type demonstrates that the visual world eye tracking paradigm used in this study was suitable. Thus, eye tracking can be used in future studies to examine referential processing in potentially even longer sign language discourses and texts. However, the current design did not reveal the predicted effect of localization. Nevertheless, as apparent from the figures in the results section, there were some minor differences between the localization conditions. There was a tendency of more looks to the object when it was overtly assigned to an R-locus with the index sign in the first sentence and a similar trend of more looks to the subject when it was overtly localized beforehand. Similarly, there seems to be a difference between match and mismatch conditions with increased target looks in the match conditions, but fewer looks in the mismatch conditions. We are aware that these trends were not significant in the analyses and should, therefore, be interpreted cautiously, but nevertheless, these differences between the conditions suggest that localization seems to do something during processing. There might be different reasons for the observed (non-)effects which are either related to the linguistic function of the index sign in DGS or to the stimulus material used in this study. In the following, we briefly discuss both options.

On the one hand, it might be the case that localization does not carry the linguistic function of increasing prominence. As already mentioned above, the overt devices can also be (simultaneously) combined with each other. For instance, the index sign may be accompanied by brow raise, body/head movement and/or eye gaze. Moreover, the index sign can also accompany a noun whose location features are adapted to the corresponding R-locus, i.e., non-body anchored signs that are produced in the neutral region of the signing space can be produced more towards the left- or righthand side to be linked to this area. At the same time, sign languages use quite different anaphoric strategies to refer back to an established discourse referent. Some of these strategies do not use spatial devices so that overt localization is again just one of the options used for reference tracking. In addition, DGS frequently uses zero forms in sentence-initial position to refer to prominent discourse referents (Nuhbalaoglu, 2018). Thus, in DGS, like in many other sign languages, overt localization of discourse referents in the signing space is only one available device for reference tracking. In combination with new discourse referents, the index sign might thus have a different function in sign languages than prominence-increasing devices (such as indefinite demonstratives in spoken languages) do. One option is that the index sign is simply used to overtly localize the corresponding discourse referent without boosting the prominence of this discourse referent in order to establish a spatial strategy of reference tracking, in line with what Frederiksen and Mayberry (2022) argue. A further difference between prominence-enhancing indefinite demonstratives in spoken languages and pointing signs in sign languages is the gestural origin of the index sign which is still visible in sign languages (McBurney, 2004; Pfau, 2011; Johnston, 2013; Cormier et al., 2013; Fenlon et al., 2019). In sign languages, pointing signs have many different functions, some of which correlate with demonstratives and pronouns. However, the degree of grammaticalization might not (yet) be sufficient to account for the very specific prominence-enhancing function of an indefinite demonstrative as described in the “Introduction” section for spoken languages such as English and German.

On the other hand, the stimulus material used for this study might have not been fully suitable for eliciting and investigating the specific discourse function of increasing prominence. Recall that we avoided any nonmanual markers (eye gaze, brow raise, head movement and/or body lean) on the antecedent since we were only interested in the effect of the overt localization introduced by the index sign. However, a bare index sign might be unnatural in this context since participants could have been expecting a morphologically more complex index sign accompanied by additional nonmanual markers. A potential alternative explanation is that, as described above, the signer was instructed to keep a longer final hold of the referent sign in case of an absent index sign. Lengthening the final hold of a sign can function as a prosodic prominence marker in DGS (Herrmann, 2015). However, we do not consider this a plausible explanation because the index signs in the first sentence were significantly longer than the final holds of the referent signs without index. While we cannot completely rule out that the final lengthening of the final hold (in the absence of an index sign) might have had an impact on our results, the index signs were thus more visually present and salient, primarily due to their longer visibility in the stimulus video.

Likewise, the form of the anaphoric expression used at the beginning of the second sentence might not have been appropriate. The repetition of a full noun anaphora to refer to an antecedent might either be referentially too strong or unexpected in this context, as for highly accessible referents reduced referential forms are preferred (Almor, 1999). In the first case, the full noun anaphora might simply overwrite the discourse effect triggered by the overt pointing in the first sentence. In the second case, a full noun anaphora might not be felicitous, also known as the *repeated name penalty* (Gordon & Hendrick, 1998) leading to changes in the processing costs that might intervene with any effect of localization. A study on hearing German-speaking children by Lehmkuhle and Schimke (2024) demonstrated that children fixated a referent more after hearing a personal pronoun than after a repeated name. Overall, this suggests fewer target looks for repeated names which is consistent with the low number of target looks observed in our study. Moreover, we already mentioned above that Frederiksen and Mayberry (2022) reported for ASL that the overt localization of a discourse referent in the first sentence boosted the use of a pronominal pointing sign in the second sentence. Therefore, we may speculate that DGS signers also expect a pointing sign in this context but not the repetition of the noun. Perhaps a comparable study with more ambiguous and reduced forms of referential expressions would reveal an effect of overt localization because the reduced forms might be more appropriate in this context and might not overwrite the localization effect. A follow-up study using different kinds of antecedents (accompanied by different additional nonmanual markers) and different kinds of pronominal expressions, e.g., index sign, eye gaze, or a null form, i.e., subject drop, instead of the full noun anaphora might provide more options for overt localization in order to investigate its effects. In principle, one would certainly expect an effect on the object referent since objects are less prominent and might therefore be affected more strongly by overt localization. Moreover, as the prominence function of the index sign might be located at the discourse/pragmatic level, the length of the discourses, i.e., two sentences, might have been too short for any potential prominence effects of localization to reveal themselves (von Heusinger & Schumacher, 2019). Future studies could increase the length of the discourse by inserting at least one, or even several, sentences before the respective referent is re-mentioned. These sentences could provide additional contextual information, which should, however, be neutral with regard to the discourse referents to avoid any bias in interpretation.

## Conclusion

This study is the first experimental study which explicitly examines the influence of overt manual localization on the accessibility of discourse referents in sign language sentence processing. Using a visual world eye tracking paradigm, we investigate whether the index sign in DGS can be used to enhance the prominence of the corresponding new discourse referent, similar to prominence-increasing devices such as indefinite demonstratives in spoken languages. The absence of localization effects in the data suggest that the study in its current form cannot clearly determine the effects of overt localization on referential processing because other competing factors at play might have intervened. We discussed two different possible explanations for why the overt localization in the first sentence did not lead to facilitatory processing of a coreferential expression in the second sentence: (i) the overt localization of new discourse referents is mainly used to initiate a spatial strategy of reference tracking, i.e., it does not carry the linguistic function of enhancing prominence, or (ii) the stimulus material used in this experimental study did not allow for revealing this specific function of localization. Follow-up studies may provide more evidence to clarify the linguistic status of referential localization and the specific discourse semantic functions of the index sign.

## Data Availability

The datasets generated during and/or analyzed in the current study as well as the code used for data analyses and data visualizations are available in the OSF repository, https://osf.io/5vubm/?view_only=e419085fd83748c5a5296318550c7109 [10.17605/OSF.IO/5VUBM].

## Footnotes

1 Notational conventions: signs are glossed in small caps. Subscripts refer to points in the signing space, i.e., ‘r’ indicates the right side of the signer. index denotes a pointing sign, typically produced by the extended index finger. Lines above a sign indicate an accompanying nonmanual marking with abbreviations specifying the marker, i.e., ‘ht’ indicates a head tilt.
